# National Reference Values of FFMI and FMI Using Body Composition Chart in Korean Adults

**DOI:** 10.3390/nu18081170

**Published:** 2026-04-08

**Authors:** Hyeoijin Kim, Yong Hee Hong, Young Charles Jang, Youngil Lee, Jae Young Lee, Seon Ho Eom, Sochung Chung, Chul-Hyun Kim

**Affiliations:** 1 Department of Physical Education, Korea National University of Education, Chungju 28173, Republic of Korea; aries408@knue.ac.kr; 2 Department of Pediatrics, Soonchunhyang University Bucheon Hospital, Soonchunhyang University College of Medicine, Bucheon 14584, Republic of Korea; dr4baby@naver.com; 3 Department of Orthopedics, School of Medicine, Emory Musculoskeletal Institute, Emory University, Atlanta, GA 30329, USA; young.jang@emory.edu; 4 Department of Movement Sciences and Health, Usha Kundu, MD College of Health, University of West Florida, Pensacola, FL 32514, USA; ylee1@uwf.edu; 5 Department of ICT Convergence, Soonchunhyang University, Asan 31538, Republic of Korea; lhsljy58@gmail.com; 6 Department of Sports Medicine, Soonchunhyang University, Asan 31538, Republic of Korea; ush1995@sch.ac.kr; 7 Department of Pediatrics, Konkuk University Medical Center, School of Medicine, Konkuk University, Seoul 05030, Republic of Korea

**Keywords:** body composition, fat-free mass index, fat mass index, body composition chart, obesity subtypes, bioelectrical impedance analysis, KNHANES, reference values, aging, sarcopenic obesity

## Abstract

**Background/Objectives**: Body mass index (BMI) cannot distinguish fat mass (FM) from fat-free mass (FFM). The fat-free mass index (FFMI = FFM/height^2^) and fat mass index (FMI = FM/height^2^) decompose BMI into lean and fat components. We aimed to establish the first nationally representative, BIA-based FFMI and FMI reference values for Korean adults, visualize body composition trajectories, and classify obesity subtypes using the body composition chart. **Methods**: Cross-sectional data from 10,140 participants (4508 men, 5632 women; aged 10–80 years) from KNHANES IX (2022–2023) were analyzed. Multifrequency BIA (InBody 970) measured FFM and FM. Obesity subtypes (%BF ≥ 25% men, ≥35% women) were classified as underlean (FFMI < P5), proportional (P5–P95), or heavy (>P95) relative to an 18–59-year-old reference. **Results**: In men, FFMI peaked at 19.1 kg/m^2^ (30–49 years), declining 9.7% by 70–80 years, while FMI remained stable. In women, FMI increased 44% with stable FFMI. Underlean obesity in men rose from 0.7% (30–39 years) to 17.4% (70–80 years), undetected by BMI or waist circumference. **Conclusions**: These reference values and body composition chart provide practical tools for identifying underlean obesity and assessing body composition beyond BMI in Korean adults.

## 1. Introduction

Body mass index (BMI), calculated as body weight divided by the square of height (kg/m^2^), has served as the cornerstone of nutritional status assessment and obesity classification for decades [1,2]. However, BMI is limited in distinguishing fat mass (FM) from fat-free mass (FFM), the two principal components of body weight [3,4]. Consequently, individuals with the same BMI may have markedly different proportions of FM and FFM, leading to potential misclassification of both obesity and malnutrition [3,5].

Fat mass comprises all adipose tissue in the body and serves primarily as an energy reserve; excess FM is associated with increased risk of cardiovascular disease, type 2 diabetes, and metabolic syndrome [6]. Fat-free mass includes skeletal muscle, bone, organs, and body water, and represents the metabolically active tissue compartment that is the primary determinant of resting metabolic rate [7]. Higher FFM is associated with greater physical function and overall survival, whereas low FFM—as seen in sarcopenia—is associated with disability, falls, prolonged hospitalization, and increased mortality [8,9]. The inability of BMI to capture the balance between these two functionally distinct compartments constitutes its most fundamental limitation.

This limitation is particularly consequential in aging populations. Progressive loss of FFM and concomitant gain of FM occur simultaneously during aging [10,11], a process that BMI fails to capture because the opposing changes may cancel each other out [6,7]. Cross-sectional studies consistently report that percent body fat increases and lean mass decreases with age, often in the absence of changes in body weight or BMI [11]. In men, the increase in percent FM is driven predominantly by reduced lean mass, whereas in women, FM accumulation is the primary contributor [11,12]. Understanding these sexually dimorphic aging trajectories requires body composition assessment methods that go beyond BMI.

To address this fundamental limitation, VanItallie et al. [7] proposed deconstructing BMI into two height-normalized indices: the fat-free mass index (FFMI = FFM/height^2^) and the fat mass index (FMI = FM/height^2^), such that BMI = FFMI + FMI. Hattori et al. [13,14] subsequently developed the body composition chart, a graphical method that plots FFMI on the *x*-axis and FMI on the *y*-axis, with superimposed diagonal lines representing constant BMI values and constant percentage body fat (%BF). This two-dimensional visualization simultaneously displays four body composition parameters and enables direct comparison of lean and fat components across groups, ages, and populations [13,14].

The body composition chart has been applied primarily in pediatric populations to characterize body composition trajectories during growth [3,15,16,17]. In adult populations, the chart has revealed that the age-related increase in BMI is driven disproportionately by FM accumulation, while FFM declines with aging [18,19]. Recently, Pratt et al. [20] established DXA-based body composition reference values and trajectories across adulthood in 10,033 European adults, demonstrating that lean mass indices peak between 30 and 39 years of age and decline progressively thereafter in both sexes. Similarly, de Souto Barreto et al. [11] identified sex-specific break points in the age–body composition relationship using DXA in the French INSPIRE cohort. However, no study has applied the body composition chart to a nationally representative adult population.

Using the FFMI–FMI framework, obesity can be further classified into three subtypes: underlean (sarcopenic) obesity, proportional obesity, and heavy (muscular) obesity, based on the relative contribution of FFM [19]. Underlean obesity, characterized by excess adiposity concurrent with depleted lean mass, is clinically important because it is associated with greater insulin resistance, functional limitation, and mortality risk compared with proportional obesity at equivalent levels of adiposity [21,22]. A recent meta-analysis estimated the global prevalence of sarcopenic obesity at 11% in older adults, with prevalence increasing substantially after age 70 [6,10,23]. The European Society for Clinical Nutrition and Metabolism (ESPEN) and the European Association for the Study of Obesity (EASO) consensus statement [22] highlighted the diagnostic challenge posed by this phenotype, as conventional BMI screening fails to identify individuals with low lean mass masked by excess fat. In pre-frail older adults, low FFMI is a stronger predictor of sarcopenia and functional decline than BMI alone [9], reinforcing the clinical value of the FFMI–FMI framework.

Body composition can be assessed by several methods, each with distinct strengths and limitations. Dual-energy X-ray absorptiometry (DXA) is considered a reference standard for clinical body composition measurement, providing precise estimates of FM, FFM, and bone mineral content [24]. However, DXA requires specialized equipment, involves low-dose radiation exposure, and is costly and less portable, limiting its applicability in large-scale population surveys. Bioelectrical impedance analysis (BIA) estimates body composition by measuring electrical impedance across body tissues and is increasingly used in epidemiological surveys due to its portability, low cost, safety, and ease of administration [25]. Modern multifrequency BIA devices, such as the InBody 970 (InBody, Seoul, Republic of Korea) used in the present study, show strong agreement with DXA for whole-body and segmental composition measures in Korean adults [18], making BIA a suitable method for deriving population-level reference values.

Several studies have established population-specific FFMI and FMI reference values, including Swiss Caucasians aged 18–98 years [26], Austrian adults from the LEAD cohort [27], Italian adults [24], Japanese older adults [28], Singaporean adults [29], and Chinese multiethnic adults [30]. These studies consistently demonstrate that reference ranges are sex-, age-, and ethnicity-specific, underscoring the need for population-appropriate normative data [24,26,28,30,31]. In Korea, we previously established the first DXA-based FFMI and FMI reference values and obesity subtypes in 1275 community-dwelling adults aged 18–89 years [19]. However, this earlier study was based on a single-center convenience sample, limiting generalizability. Subsequent KNHANES IV–V DXA studies provided nationally representative body composition curves [3,32,33,34], but did not include BIA measurements and were collected over a decade ago.

The KNHANES IX (2022–2023) introduced multifrequency BIA (InBody 970) for the first time, providing whole-body and segmental composition data in a nationally representative sample of Korean participants aged 10–80 years, spanning adolescence through old age [35]. To date, no study has utilized these data to establish FFMI and FMI reference values, apply the body composition chart, or characterize obesity subtypes at the national level. Therefore, the objectives of this study were (1) to establish the first nationally representative BIA-based FFMI and FMI reference values for Korean adults; (2) to visualize sexually dimorphic body composition trajectories using the body composition chart; and (3) to classify and characterize obesity subtypes and their age-dependent patterns using the combined FFMI–FMI framework.

Based on prior evidence from European [12,20,26] and Japanese [28] populations, we hypothesized that (1) Korean men would exhibit a predominantly lean mass-driven BMI decline with aging (leftward trajectory on the body composition chart), whereas women would show a predominantly fat mass-driven BMI increase (upward trajectory); and (2) the prevalence of underlean obesity would increase with age in men due to progressive sarcopenia, while remaining low in women due to relative FFMI preservation.

## 2. Materials and Methods

### 2.1. Study Design and Population

This cross-sectional study used data from the KNHANES IX, 1st (2022) and 2nd (2023), conducted by the Korea Disease Control and Prevention Agency (KDCA). The KNHANES employs a complex, stratified, multistage probability sampling design targeting the non-institutionalized civilian population [35]. In each survey year, approximately 192 primary sampling units were selected from across the country, with 4800 households and approximately 10,000 individuals aged ≥1 year sampled. All examinations were conducted at Mobile Examination Centers (MECs)—specially equipped vehicles that travel to sampled districts—where trained health professionals performed standardized anthropometric measurements, BIA assessments, blood sampling, and questionnaire surveys in a single visit [35].

To determine the sample from a total of 13,194 participants (2022: n = 6265; 2023: n = 6929), the following inclusion criteria were applied: (1) aged 10–80 years; (2) valid BIA measurement (excluding missing codes 888 [impossible value] and 999 [non-response]); (3) valid height measurement; and (4) non-zero survey weight. Exclusion criteria were (1) extracellular water-to-total body water ratio (ECW/TBW) ≥0.40, indicating suspected edema or fluid imbalance that compromises BIA measurement accuracy [35]; and (2) current pregnancy (HE_prg = 1). The final analytic sample comprised 10,140 participants (4508 men, 5632 women). A participant flow diagram is provided in Supplementary Figure S1.

Because KNHANES is designed to be representative of the non-institutionalized civilian population of Korea, participants with chronic diseases (e.g., diabetes, hypertension, cancer, thyroid disease, chronic kidney disease) were not excluded from the analysis. This general population approach is consistent with previous KNHANES-based body composition reference studies [32,33,34] and NHANES-based reference analyses. It ensures that the resulting reference values reflect the full distribution of body composition in the general Korean adult population, including individuals with common chronic conditions. This approach differs from “healthy population” reference studies that exclude individuals with self-reported diseases [26], which may yield reference values that are not directly applicable to the general population encountered in clinical and public health practice.

The study protocol was approved by the Institutional Review Board of Soonchunhyang University (IRB No. 1040875-2026-03-004). As this study involved secondary analysis of publicly available, de-identified data from the Korea National Health and Nutrition Examination Survey (KNHANES), the requirement for informed consent was waived in accordance with the National Health Promotion Act, Article 16.

### 2.2. Anthropometric Measurements

Height was measured to the nearest 0.1 cm (SECA 225, Secal GmbH, Hamburg, Germany) and weight to the nearest 0.1 kg (GL-6000-20, CASKOREA, Seoul, Republic of Korea). BMI was classified per the Korean Society for the Study of Obesity (KSSO) 2023 criteria: underweight (<18.5), normal (18.5–22.9), overweight (23.0–24.9), class I obesity (25.0–29.9), class II obesity (30.0–34.9), and class III obesity (≥35.0 kg/m^2^) [2]. Waist circumference was measured at the midpoint between the lowest rib and the iliac crest.

### 2.3. Body Composition Assessment

Whole-body and segmental body composition were measured by multifrequency BIA, using a direct segmental multifrequency device (InBody 970, InBody Co., Seoul, Republic of Korea) operating at six frequencies (1, 5, 50, 250, 500, and 1000 kHz) with eight tactile electrodes placed on both hands and feet. Variables obtained included fat-free mass (FFM; kg, representing total body FFM), body fat mass (BFM; kg), percent body fat (%BF), total body water (TBW), and extracellular water (ECW).

Participants with ECW/TBW ≥ 0.40 were excluded from the primary analysis, as an ECW/TBW ratio at or above this threshold indicates fluid overload or edema, which alters tissue conductivity and reduces the reliability of BIA-derived body composition estimates [35]. This threshold is consistent with the KNHANES user guide recommendations and has been applied in prior BIA studies to ensure measurement quality [35].

To ensure nationally representative estimates, all analyses incorporated survey weights, as recommended by the KDCA. The KNHANES uses complex, stratified, multistage probability sampling; applying appropriate survey weights, along with the stratification variable (kstrata) and primary sampling unit (psu), accounts for the unequal selection probabilities and the multistage design [35]. For 2022 data, the BIA-specific weight variable (wt_bia) was used. For 2023 data, the interview and examination weight variable (wt_itvex) was used, as it provides complete coverage of all participants with valid BIA measurements [35]. When pooling 2022 and 2023 data, weights were divided by two, as recommended by the KDCA for multi-year analyses.

### 2.4. Calculation of Body Composition Indices

FFMI and FMI were calculated as FFM and BFM divided by height squared (m^2^), respectively, such that BMI = FFMI + FMI [7]. This identity holds because FFM and BFM sum to total body weight, and dividing by height^2^ preserves the additivity.

### 2.5. Body Composition Chart Construction

The body composition chart was constructed by plotting FFMI (*x*-axis) against FMI (*y*-axis), following Hattori et al. [13,14]. Diagonal lines for constant BMI (18.5, 23, 25, 30, and 35 kg/m^2^) were superimposed, as were lines for constant %BF (0%, 15%, 25%, 35%). Mean FFMI and FMI by sex and age group were plotted as chronologically connected trajectory points. A 10% random subsample was displayed as background scatter to visualize the full distribution.

### 2.6. Reference Values and Percentile Construction

Sex- and age-group-specific percentiles (5th, 10th, 25th, 50th, 75th, 90th, and 95th) were calculated for FFMI and FMI with appropriate survey weighting (linearization variance estimation). The following age groups were used: 10–19, 20–29, 30–39, 40–49, 50–59, 60–69, and 70–80 years. Smoothed percentile curves were generated using a 5-year moving window across single years of age, a method that balances smoothness against age-specific precision while preserving the overall shape of the distribution [13].

### 2.7. Obesity Subtype Classification

Obesity was defined as %BF ≥ 25% (men) and ≥35% (women), based on the widely adopted healthy %BF ranges established by Gallagher et al. [36], using a multiethnic sample and a four-compartment model for validation.

Among participants classified as obese, three subtypes were defined based on FFMI relative to the 18–59-year reference group [19]. Underlean obesity was defined as FFMI below the 5th percentile (men: <16.0 kg/m^2^; women: <13.2 kg/m^2^), indicating depleted lean mass despite excess adiposity. Heavy obesity was defined as FFMI above the 95th percentile (men: >22.2 kg/m^2^; women: >18.5 kg/m^2^), indicating excess lean mass. Proportional obesity comprised the remainder (FFMI between P5 and P95). These thresholds were derived from the nationally representative 18–59-year subgroup of the present study and are consistent with those reported in our prior DXA-based study [19].

### 2.8. BMI Decomposition Analysis

Mean FFMI, FMI, and the FFMI-to-BMI ratio (FFMI/BMI × 100%) were calculated for each BMI category in adults ≥19 years to quantify the heterogeneity of body composition within BMI strata. BMI categories followed the Korean Society for the Study of Obesity (KSSO) 2023 criteria [2]: underweight (<18.5 kg/m^2^), normal weight (18.5–22.9 kg/m^2^), overweight (23.0–24.9 kg/m^2^), class I obesity (25.0–29.9 kg/m^2^), class II obesity (30.0–34.9 kg/m^2^), and class III obesity (≥35.0 kg/m^2^).

### 2.9. Statistical Analysis

All analyses incorporated the complex survey design using the stratification variable (kstrata), primary sampling unit (psu), and year-specific sampling weights (2022: wt_bia; 2023: wt_oe) with linearization-based variance estimation to account for the multistage probability sampling design of KNHANES. Missing values for BIA variables were coded as 888 (impossible values) or 999 (non-response) and replaced with missing values prior to analysis. DXA variables were not used in this study due to the age restriction of ≥40 years in KNHANES IX, 3rd (2024).

Descriptive statistics and between-group comparisons were performed using IBM SPSS Statistics version 26.0 (IBM Corp., Armonk, NY, USA). Survey-weighted means ± standard deviation (SD) were computed for all continuous variables using the complex samples module, and between-sex and between-age-group differences in FFMI and FMI were tested using the complex samples general linear model. Categorical variables are reported as weighted percentages with 95% confidence intervals; statistical significance was defined as a two-tailed *p* < 0.05.

Advanced analyses, including survey-weighted percentile estimation, body composition chart construction, smoothed percentile curve generation using a 5-year moving window, obesity subtype classification, and BMI decomposition, were performed in Python 3.12 (Python Software Foundation, Wilmington, DE, USA) using the following packages: NumPy 2.4, pandas 3.0, statsmodels 0.14, and matplotlib 3.10. Differences in body composition variables among obesity subtypes were assessed using survey-weighted one-way analysis with cluster-robust standard errors, followed by Bonferroni-corrected pairwise comparisons. Different superscript letters within each sex denote statistically significant pairwise differences (*p* < 0.05). Continuous variables without a normal distribution are presented as unweighted medians with interquartile ranges.

### 2.10. Use of Cenerative AI Tools

A generative AI tool (Claude, Anthropic, Claude Opus 4, San Francisco, CA, USA) was used to assist with Python code generation and execution for statistical analysis and figure creation, and table formatting. All AI-assisted outputs were independently reviewed and verified by the authors, who take full responsibility for the content of this publication.

## 3. Results

### 3.1. Participant Characteristics

Table 1 presents the characteristics of 10,140 participants by sex and age group. In men, the mean FFMI peaked at 19.1 kg/m^2^ (30–49 years) and declined to 17.2 kg/m^2^ by 70–80 years (−9.7%), and the mean FMI in men was stable across adult ages (6.0–6.5 kg/m^2^). This pattern indicates that the BMI decline observed in older men is driven predominantly by lean mass loss rather than reduced adiposity. In women, FFMI increased gradually to 15.8 kg/m^2^ (60–69 years) before declining to 15.5 kg/m^2^ (70–80 years, a decline of 1.9%). Also, the FMI in women increased steadily from 6.0 kg/m^2^ (10–19 years) to 8.6 kg/m^2^ (70–80 years), representing a 44% increase from the adolescent baseline. In contrast to men, the increase in BMI with aging in women reflects fat mass accumulation superimposed on a relatively preserved lean mass compartment.

### 3.2. FFMI and FMI Percentile Reference Values

**Figure 1:**
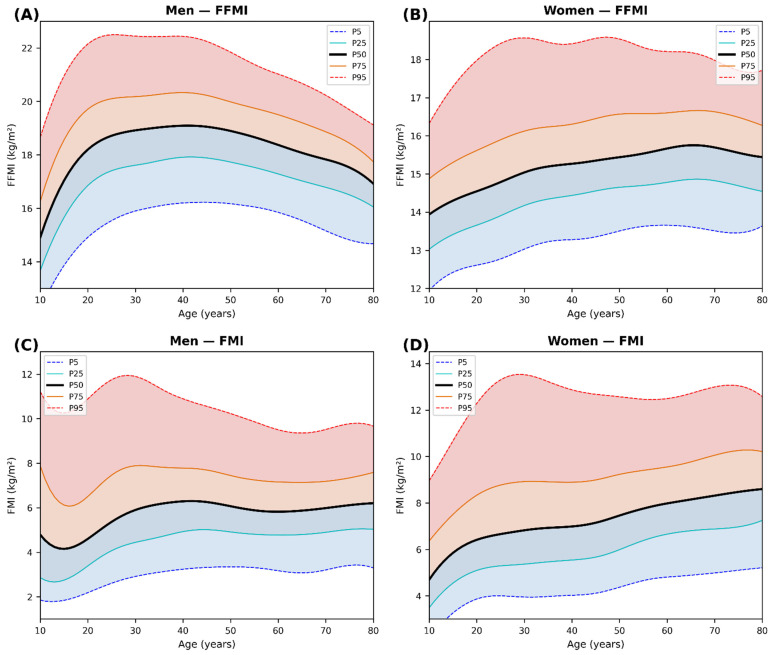
Smoothed sex-specific percentile curves (P5, P10, P25, P50, P75, P90, P95) for fat-free mass index (FFMI) and fat mass index (FMI) from age 10 to 80 years, derived from KNHANES IX, 1st (2022) and 2nd (2023). Survey-weighted 5-year moving window smoothing applied. (**A**) Men FFMI; (**B**) Women FFMI; (**C**) Men FMI; (**D**) Women FMI.

Table 2 presents sex- and age-specific percentiles. Two overarching patterns emerged. First, FFMI showed a marked sex difference in age-related trajectory: in men, median FFMI peaked at 30–39 years (19.0 kg/m^2^) and declined to 17.3 kg/m^2^ by 70–80 years, whereas in women, median FFMI was notably stable across adult ages (14.7–15.8 kg/m^2^). Second, FMI showed a more pronounced age-related increase in women (median: 5.6–8.5 kg/m^2^) than in men (4.4–6.3 kg/m^2^), indicating that fat accumulation is the primary driver of BMI increase in aging women. The percentile distributions (Figure 1A–D) widened with age in both sexes, reflecting increasing inter-individual variability in body composition during aging.

Smoothed percentile curves (Figure 1A,B for FFMI; Figure 1C,D for FMI) illustrate these trajectories continuously from 10 to 80 years of age. Reference values for the 18–59-year group were determined as follows: for FFMI, the P5–P95 range was 16.0–22.2 kg/m^2^ in men and 13.2–18.5 kg/m^2^ in women, and for FMI, the P5–P95 range was 3.0–10.8 kg/m^2^ in men and 4.2–12.9 kg/m^2^ in women.

### 3.3. Body Composition Chart Analysis

**Figure 2:**
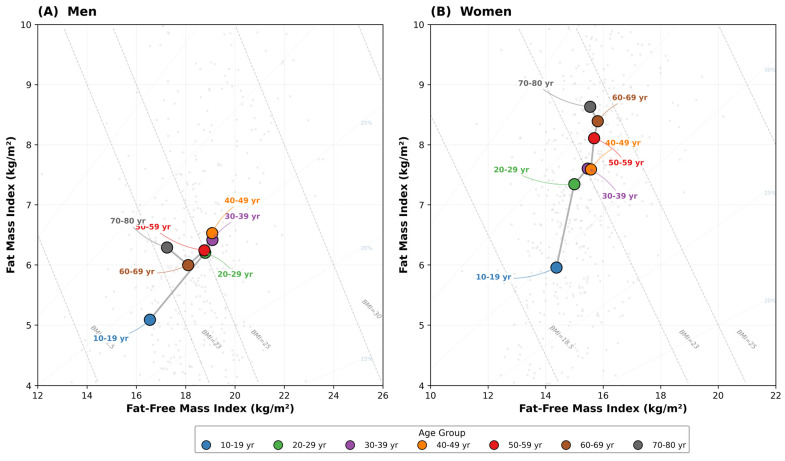
Body composition chart plotting fat-free mass index (FFMI, *x*-axis) against fat mass index (FMI, *y*-axis) for Korean adults by sex. Superimposed diagonal lines indicate constant BMI values (18.5, 23, 25, 30, and 35 kg/m^2^) and constant percent body fat (%BF). Filled circles represent age-group mean values, arranged chronologically; arrows indicate the direction of the aging trajectory. Background scatter shows a 10% random subsample. (**A**) Men; (**B**) Women.

Figure 2 presents the body composition chart with age-group mean trajectories for men (Figure 2A) and women (Figure 2B). In men, the trajectory showed rightward FFMI expansion from adolescence to young adulthood (10–19 years: FFMI 16.6, FMI 5.1 kg/m^2^ → 30–39 years: FFMI 19.1, FMI 6.4 kg/m^2^), followed by progressive leftward displacement with stable FMI throughout older age (70–80 years: FFMI 17.2, FMI 6.3 kg/m^2^). In women, both FFMI and FMI increased modestly with middle age. Beyond 50 years, FFMI plateaued, while FMI continued to increase, resulting in predominantly upward displacement. These trajectories confirm the a priori hypothesis of sexually dimorphic aging patterns: the BMI decline in older men is driven by lean mass loss, while the BMI increase in older women is driven almost entirely by fat mass accumulation.

### 3.4. Obesity Subtypes

Table 3 presents obesity subtypes. Overall, 44.7% of men and 36.1% of women had obesity (%BF-defined). Survey-weighted ANOVA confirmed significant differences among the four groups (non-obese, underlean, proportional, heavy) for all variables in both sexes (all *p* < 0.001). Bonferroni-corrected pairwise comparisons are indicated by superscript letters in Table 3, Part A. In men, underlean obesity increased from 0.7% (30–39 years) to 17.4% (70–80 years), while heavy obesity decreased from 9.3% to 0.0% (Table 3 Part B). Individuals with underlean obesity had similar %BF to those with proportional obesity (men: 30.7% vs. 29.6%; women: 37.5% vs. 38.8%) but substantially lower BMI (men: 21.8 vs. 26.9 kg/m^2^; women: 20.4 vs. 26.1 kg/m^2^), indicating obesity concealed by low lean mass. Notably, underlean obese men had waist circumferences similar to those of non-obese men (80.6 vs. 81.4 cm, *p* > 0.05), demonstrating that this phenotype evades not only BMI screening but also waist circumference-based assessment. In women, underlean obesity was rare (0.9%), consistent with the relative preservation of FFMI observed in the percentile analysis (Section 3.2).

### 3.5. BMI Decomposition

**Figure 3:**
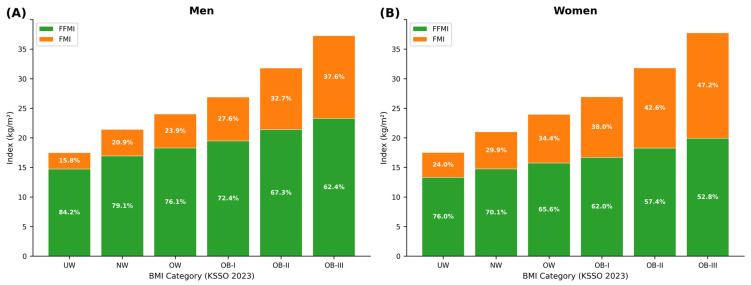
BMI decomposition into fat-free mass index (FFMI) and fat mass index (FMI) by BMI category. Bar segments represent mean FFMI (lower, filled) and FMI (upper, hatched). Numbers above bars indicate the FFMI-to-BMI ratio (%). (**A**) Men; (**B**) Women. BMI categories defined by KSSO 2023 criteria [2].

Figure 3 presents BMI decomposition by KSSO 2023 category for men (Figure 3A) and women (Figure 3B). In men, the FFMI-to-BMI ratio decreased progressively from 83.8% (underweight) to 62.4% (class III obesity), indicating that higher BMI categories are increasingly driven by fat mass rather than lean mass. The corresponding FMI-to-BMI ratio increased from 16.2% to 37.6%. In women, the same pattern was observed, with the FFMI-to-BMI ratio declining from 76.0% (underweight) to 52.7% (class III obesity) and the FMI proportion rising from 24.0% to 47.3%. Notably, even within the normal BMI category, the FMI contribution differed substantially between the sexes (men: 21.0% vs. women: 29.9%), reflecting the well-established sex difference in body fat at equivalent BMI.

### 3.6. Visualization of Obesity Subtypes on the Body Composition Chart

**Figure 4:**
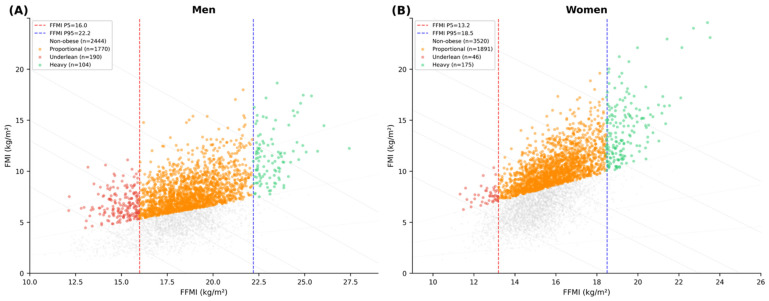
Comparison of obesity subtypes (underlean, proportional, heavy) across age groups by sex. Underlean obesity is defined as an FFMI < P5 for the 18–59-year reference group (men: P5 = 16.0 kg/m^2^; women: P5 = 13.2 kg/m^2^). Heavy obesity is defined as FFMI > P95 (men: P95 = 22.2; women: P95 = 18.5 kg/m^2^). Proportional obesity = remainder. Obesity is defined as %BF ≥ 25% (men) or ≥35% (women) [28]. (**A**) Men; (**B**) Women.

Figure 4 displays the distribution of obesity subtypes on the body composition chart for men (Figure 4A) and women (Figure 4B), with FFMI P5 and P95 of the 18–59-year reference group indicated by vertical dashed lines. In men, underlean obese individuals (n = 137) clustered to the left of the P5 boundary (FFMI < 16.0 kg/m^2^) with elevated FMI, occupying a distinct region of low FFMI and moderate-to-high FMI that overlaps with the BMI normal range. Proportional obese men (n = 1683) formed a dense central cluster between the P5 and P95 lines, whereas heavy obese men (n = 94) dispersed rightward beyond the P95 boundary (FFMI > 22.2 kg/m^2^) at varying levels of FMI. In women, the overall distribution was shifted upward relative to men, reflecting higher FMI at comparable FFMI values. Underlean obese women (n = 50) similarly appeared to the left of the P5 boundary (FFMI < 13.2 kg/m^2^), while heavy obese women (n = 166) extended beyond the P95 boundary (FFMI > 18.5 kg/m^2^) with substantially elevated FMI. The body composition chart thus reveals that underlean and proportional obese individuals are indistinguishable by BMI alone, as both groups occupy overlapping BMI isolines, whereas the chart clearly separates them along the FFMI axis.

## 4. Discussion

For the first time in the literature, this study established nationally representative, BIA-based FFMI and FMI reference values for Korean adults, applied the body composition chart to a national population, and characterized obesity subtypes across the adult lifespan.

### 4.1. Clinical Significance of FFMI and FMI Reference Values

The percentile reference values fill a critical gap in the Korean body composition literature. Prior data were derived from single-center DXA studies [19] or KNHANES IV–V DXA data without BIA [32,33,34]. The present study provides the first nationally representative, BIA-based FFMI and FMI percentiles for Korean adults aged 10–80 years, complementing existing DXA-based reference values from European [24,35], Asian [28,29], and multiethnic [30] populations.

The clinical utility of these reference values can be illustrated with a specific example. A 72-year-old Korean man with BMI 23.0 kg/m^2^—classified as “normal weight” by KSSO criteria—but FFMI 14.5 kg/m^2^ (below the age-specific 5th percentile of 14.7 kg/m^2^) and FMI 8.5 kg/m^2^ would be classified as underlean obese: a high-risk phenotype associated with functional decline, insulin resistance, and increased mortality [9,21]. This individual would be entirely missed by BMI or waist circumference screening. The FFMI–FMI framework and the reference values presented here enable clinicians to identify such individuals and initiate targeted interventions, including resistance exercise and protein supplementation [22]. Furthermore, low FFMI has been identified as an independent predictor of all-cause mortality in middle-aged and older adults, reinforcing the prognostic value of these reference values beyond nutritional screening alone.

The sex-specific patterns observed here deserve clinical attention. In men, FFMI declined by 9.7% from its peak (30–49 years) to 70–80 years of age, reflecting well-documented age-related muscle loss [8,35]. This rate of decline is consistent with findings from the INSPIRE cohort, where DXA-derived lean mass indices showed significant break points in the fifth decade of life in men [11], and with the findings of Pratt et al., who reported that appendicular lean mass peaked at 30–39 years of age and declined progressively thereafter in European men [20]. In women, FFMI continued to increase throughout their 60s before declining by only 1.9%, a pattern consistent with the combined effects of height loss in older age [26] and postmenopausal hormonal changes that promote relative preservation of lean mass alongside fat accumulation [11,37]. Korean values were lower than Swiss Caucasian references [34] but similar to those of Japanese older adults [28] and Singaporean adults [29], supporting the necessity of ethnicity-specific norms.

### 4.2. Sexually Dimorphic Aging Trajectories on the Body Composition Chart

The body composition chart provided unique insights into sexually dimorphic aging patterns concealed by BMI. In men, the trajectory traced a leftward retreat (declining FFMI) with stable FMI, indicating that BMI decline in older men reflects lean mass loss, not reduced adiposity. In women, the predominantly upward trajectory (increasing FMI) with stable FFMI indicates that BMI increase reflects fat accumulation. These findings confirm our a priori hypotheses and are consistent with observations among German adults by Bosy-Westphal and Müller [12] and those in the French INSPIRE cohort [11].

These trajectories have distinct public health implications. In men, the progressive leftward displacement on the body composition chart beginning in the fourth decade supports early initiation of muscle-preserving interventions, including resistance training and adequate protein intake. In women, the predominantly upward displacement—particularly accelerating after menopause—calls for fat mass management strategies targeting this critical window. The body composition chart thus provides a visual framework for translating population-level trajectory data into sex- and age-specific clinical recommendations.

To our knowledge, this is the first application of a body composition chart to a nationally representative adult population. Previous applications have been limited to pediatric populations [3,15,16,17], single-center lifespan studies [12,18], and one small elderly Indian cohort [22].

### 4.3. Obesity Subtypes and Clinical Implications

The age-dependent shift in obesity subtypes was striking. In men, underlean obesity increased from 0.7% (30–39 years) to 17.4% (70–80 years), while heavy obesity declined from 9.3% to 0.0%. Survey-weighted ANOVA with Bonferroni-corrected pairwise comparisons confirmed that all three obesity subtypes differed significantly in BMI, %BF, FFMI, FMI, and waist circumference (all *p* < 0.001). Notably, underlean obese men had waist circumferences similar to those of non-obese men (80.6 vs. 81.4 cm, *p* > 0.05), demonstrating that this phenotype evades not only BMI screening but also waist circumference screening.

Underlean obesity is clinically concerning because it represents convergent excess fat and deficient lean mass, a phenotype closely related to sarcopenic obesity [21,22]. A recent meta-analysis estimated the global prevalence of sarcopenic obesity at 11% in older adults [6], with more recent data suggesting even higher prevalence rates when standardized ESPEN–EASO diagnostic criteria are applied [23]. The ESPEN–EASO consensus [22] has highlighted the diagnostic challenge of identifying sarcopenic obesity using conventional anthropometric measures, and the FFMI–FMI framework offers a practical clinical identification approach that does not require DXA.

The body composition chart provides a direct visual demonstration of this diagnostic challenge. On the chart, underlean obese individuals clustered to the left of the FFMI P5 boundary with elevated FMI, occupying a region that substantially overlaps with the non-obese distribution along the BMI isolines. This overlap confirms that underlean obesity cannot be separated from normal-weight individuals using BMI alone. By contrast, the chart clearly separates underlean, proportional, and heavy obese individuals along the FFMI axis, with each subtype occupying a visually distinct region. The chart thus serves a dual diagnostic function: it simultaneously reveals the degree of adiposity (FMI axis) and the adequacy of lean mass (FFMI axis), enabling subtype-specific clinical assessment.

The rarity of underlean obesity in women (0.9% overall, compared with 3.5% in men) was consistent across the 14-year interval between our prior DXA-based study [19] and the present BIA-based analysis. This persistent sex difference suggests a genuine biological phenomenon likely related to the relative preservation of FFM in women through the hormonal milieu of the premenopausal period and the lower absolute rate of age-related muscle loss in women compared with men [11] rather than a methodological artifact of the measurement modality.

### 4.4. BMI Decomposition

The FFMI-to-BMI ratio decreased from 84% (underweight) to 62% (class III obesity) in men, extending the framework of VanItallie et al. [7] and Wells [3] to Korean adults. The substantial FFMI variability within normal BMI (SD = 1.1 kg/m^2^ in men) demonstrates that two men with identical BMI may differ by up to 7 kg/m^2^ in lean mass, a finding with direct clinical relevance for the interpretation of BMI in both nutritional and obesity assessment settings [8,9,21].

### 4.5. Strengths and Limitations

This study has several strengths. First, it provides the first nationally representative BIA-based FFMI and FMI reference values for Korean adults. Second, it represents the first application of the body composition chart to a national adult population. Third, the large sample size (n = 10,140) with appropriate complex survey weighting ensures population-level representativeness. Fourth, the close agreement between the present nationally representative BIA-based FFMI reference values (2022–2023) and the prior single-center DXA-based values from 2011 [19] with P5–P95 ranges differing by less than 0.5 kg/m^2^ provides cross-modal validation and suggests that these reference ranges reflect the true population distribution rather than modality-specific artifacts. However, the observed differences may also partly reflect secular trends in body composition over this 14-year period.

This study also has several limitations. First, the cross-sectional design precludes causal inference about aging trajectories. Second, BIA accuracy is influenced by hydration status and device-specific algorithms [25]. Third, the concurrent DXA data from KNHANES IX, 3rd (2024), were not incorporated due to the age restriction (≥40 years) and the planned companion paper. Fourth, no four-compartment model validation for the InBody 970 in Korean adults exists, precluding assessment of absolute accuracy against criterion methods. Fifth, the inclusion of participants aged 10–19 years, while useful for capturing the adolescent-to-adult transition trajectory, means that the youngest age group includes growing individuals whose body composition is still changing; the reference values for this group should be interpreted with this consideration. Sixth, participants with chronic diseases known to affect body composition (e.g., cancer cachexia, thyroid dysfunction, chronic kidney disease) were not excluded, as the study aimed to provide general population reference values representative of the Korean civilian population. These conditions may influence individual body composition values, and clinicians should consider disease status when interpreting an individual’s position relative to these reference percentiles.

Future directions include application to mortality and morbidity cohort data [38], establishment of appendicular lean mass index reference values alongside FFMI for sarcopenia screening [39], validation of the underlean obesity phenotype against clinical outcomes such as disability, hospitalization, and mortality, and secular trend analysis using forthcoming KNHANES X data.

## 5. Conclusions

This study established the first nationally representative, BIA-based FFMI and FMI reference values for Korean adults aged 10–80 years. The body composition chart revealed sexually dimorphic aging trajectories: men exhibit predominantly lean mass loss (leftward displacement), while women predominantly show fat mass gain (upward displacement). Underlean obesity increased markedly with age in men (17.4% at 70–80 years), remaining undetectable by BMI or waist circumference screening. BMI decomposition quantified the progressive shift from a lean- to fat-dominated body composition across BMI categories. These findings support the integration of FFMI and FMI into routine clinical assessments, particularly for identifying underlean obesity in older men. The reference values and body composition chart framework presented here provide practical, BIA-based tools for clinicians and public health practitioners to assess body composition beyond BMI in Korean adults.

## Figures and Tables

**Table 1:** Participant characteristics by sex and age group (mean ± SD).

Age Group (years)	n	Age (years)	Height (cm)	Weight (kg)	BMI (kg/m^2^)	%BF (%)	FFMI (kg/m^2^)	FMI (kg/m^2^)	WC (cm)
**Men**									
10–19	494	13.9 ± 2.9	164.4 ± 13.0	59.4 ± 17.0	21.7 ± 4.4	22.3 ± 8.5	16.6 ± 2.4	5.1 ± 2.8	74.2 ± 11.8
20–29	472	24.9 ± 2.6	174.8 ± 6.3	76.4 ± 15.3	24.9 ± 4.6	23.7 ± 7.2	18.8 ± 2.1	6.2 ± 3.0	85.5 ± 11.6
30–39	515	34.7 ± 3.0	175.3 ± 5.5	78.4 ± 13.2	25.5 ± 3.9	24.4 ± 6.2	19.1 ± 1.9	6.4 ± 2.5	88.7 ± 10.3
40–49	668	44.5 ± 2.9	173.8 ± 6.0	77.6 ± 12.4	25.6 ± 3.7	24.9 ± 5.7	19.1 ± 1.8	6.6 ± 2.3	90.1 ± 9.8
50–59	712	54.5 ± 2.9	170.5 ± 5.6	72.8 ± 10.5	25.0 ± 3.3	24.5 ± 5.3	18.8 ± 1.7	6.3 ± 2.1	89.2 ± 8.7
60–69	911	64.5 ± 2.8	168.4 ± 5.7	68.6 ± 9.9	24.1 ± 3.0	24.4 ± 5.3	18.1 ± 1.6	6.0 ± 1.9	88.0 ± 8.7
70–80	736	75.4 ± 3.4	165.8 ± 5.6	64.9 ± 9.0	23.6 ± 2.8	26.1 ± 5.5	17.3 ± 1.5	6.3 ± 1.9	88.2 ± 8.6
**Total**	**4508**	**48.6 ± 20.0**	**170.1 ± 7.9**	**70.9 ± 13.7**	**24.4 ± 3.8**	**24.5 ± 6.2**	**18.2 ± 2.0**	**6.1 ± 2.3**	**86.9 ± 10.8**
**Women**									
10–19	470	14.0 ± 2.9	157.4 ± 8.3	50.7 ± 11.5	20.3 ± 3.6	28.3 ± 6.7	14.4 ± 1.5	6.0 ± 2.4	67.6 ± 8.6
20–29	542	24.6 ± 2.8	161.8 ± 5.6	58.5 ± 12.3	22.3 ± 4.4	31.8 ± 6.6	15.0 ± 1.7	7.3 ± 3.1	73.1 ± 10.4
30–39	631	34.8 ± 2.8	162.0 ± 5.2	60.3 ± 11.3	23.0 ± 4.3	31.8 ± 6.8	15.4 ± 1.6	7.6 ± 3.0	76.8 ± 10.2
40–49	924	44.4 ± 2.9	161.2 ± 5.4	60.2 ± 10.8	23.2 ± 4.0	31.9 ± 6.3	15.6 ± 1.6	7.6 ± 2.8	78.1 ± 10.1
50–59	1047	54.4 ± 2.8	158.1 ± 5.1	59.5 ± 9.1	23.8 ± 3.5	33.4 ± 5.6	15.7 ± 1.5	8.1 ± 2.4	81.0 ± 9.2
60–69	1189	64.3 ± 2.8	155.7 ± 5.1	58.7 ± 8.7	24.2 ± 3.4	34.0 ± 5.5	15.8 ± 1.4	8.4 ± 2.4	83.2 ± 9.0
70–80	829	75.0 ± 3.4	152.3 ± 5.5	56.1 ± 8.4	24.2 ± 3.3	35.0 ± 5.9	15.6 ± 1.3	8.6 ± 2.4	84.9 ± 8.9
**Total**	**5632**	**49.5 ± 18.7**	**158.0 ± 6.5**	**58.2 ± 10.4**	**23.3 ± 3.9**	**32.8 ± 6.3**	**15.5 ± 1.5**	**7.8 ± 2.7**	**79.2 ± 10.7**

BMI, body mass index; %BF, percent body fat; FFMI, fat-free mass index; FMI, fat mass index; WC, waist circumference; SD, standard deviation.

**Table 2:** FFMI and FMI percentile reference values by sex and age group (kg/m^2^).

Age Group	P5	P10	P25	P50	P75	P90	P95	Age Group	P5	P10	P25	P50	P75	P90	P95
**Men—F** **F** **MI (kg/m^2^)**								**Women—F** **F** **MI (kg/m^2^)**							
10–19	12.9	13.5	14.7	**16.4**	18.1	19.7	20.9	10–19	12.3	12.6	13.4	**14.2**	15.2	16.5	17.1
20–29	15.5	16.3	17.4	**18.6**	20.0	21.5	22.4	20–29	12.7	13.1	13.8	**14.7**	15.8	17.1	18.2
30–39	16.1	16.7	17.8	**19.0**	20.3	21.5	22.5	30–39	13.3	13.6	14.3	**15.2**	16.2	17.5	18.6
40–49	16.2	16.7	17.9	**19.0**	20.2	21.5	22.4	40–49	13.3	13.7	14.6	**15.4**	16.4	17.8	18.6
50–59	16.1	16.7	17.6	**18.7**	19.8	20.7	21.5	50–59	13.6	14.0	14.7	**15.5**	16.6	17.6	18.3
60–69	15.6	16.0	17.0	**18.1**	19.3	20.1	20.6	60–69	13.6	14.1	14.9	**15.8**	16.6	17.6	18.2
70–80	14.8	15.4	16.3	**17.4**	18.4	19.2	19.6	70–80	13.5	14.0	14.6	**15.5**	16.5	17.2	17.7
**Men—FMI (kg/m^2^)**								**Women—FMI (kg/m^2^)**							
10–19	1.8	2.1	2.8	**4.4**	6.4	9.0	10.6	10–19	2.7	3.3	4.3	**5.6**	7.1	9.2	10.3
20–29	2.5	3.0	4.1	**5.4**	7.7	10.0	12.0	20–29	4.0	4.4	5.2	**6.5**	8.6	11.1	13.0
30–39	3.0	3.6	4.6	**6.0**	7.7	9.8	11.4	30–39	4.0	4.4	5.4	**6.9**	8.9	11.5	13.3
40–49	3.3	3.9	5.0	**6.3**	7.8	9.5	10.8	40–49	4.1	4.6	5.7	**7.1**	8.9	11.0	12.7
50–59	3.4	4.1	4.9	**6.0**	7.4	8.9	10.1	50–59	4.7	5.3	6.5	**7.8**	9.5	11.1	12.5
60–69	3.1	3.7	4.8	**5.9**	7.1	8.4	9.3	60–69	4.8	5.5	6.8	**8.1**	9.7	11.4	12.6
70–80	3.2	3.9	5.0	**6.1**	7.4	8.9	9.7	70–80	5.0	5.8	7.0	**8.5**	10.2	11.7	12.8

FFMI, fat-free mass index; FMI, fat mass index; P, percentile; Data: KNHANES IX, 1st (2022) + 2nd (2023). Reference group for obesity subtype classification (18–59 years): Men FFMI P5 = 16.0, P95 = 22.2 kg/m^2^; Women FFMI P5 = 13.2, P95 = 18.5 kg/m^2^ [19].

**Table 3:** Prevalence and characteristics of obesity subtypes by sex.

***Part A. Overall obesity subtype characteristics (mean ± SD)***										
**Sex**	**Subtype**	**n**	**Prevalence** **(%)**	**Age** **(Years)**	**BMI** **(kg/m^2^)**	**%BF** **(%)**	**FFMI** **(kg/m^2^)**	**FMI** **(kg/m^2^)**	**WC** **(cm)**	***p***
***Men***	**Non-obese**	2444	55.3	43.0 ± 18.2 ^a^	22.7 ± 2.8 ^a^	19.9 ± 3.8 ^a^	18.1 ± 1.9 ^a^	4.6 ± 1.2 ^a^	81.4 ± 8.6 ^a^	<0.001 ^†^
	Underlean	196	3.5	42.9 ± 28.0 ^a,b^	21.8 ± 1.6 ^b^	30.7 ± 4.2 ^b^	15.0 ± 0.8 ^b^	6.7 ± 1.3 ^b^	80.6 ± 8.1 ^a^	
	Proportional	1765	38.3	48.0 ± 17.5 ^b^	26.9 ± 2.8 ^c^	29.6 ± 3.7 ^c^	18.9 ± 1.5 ^c^	8.0 ± 1.7 ^c^	94.0 ± 7.3 ^b^	
	Heavy	103	2.9	35.7 ± 12.1 ^c^	34.8 ± 3.0 ^d^	32.8 ± 4.6 ^d^	23.2 ± 0.9 ^d^	11.5 ± 2.6 ^d^	109.6 ± 7.8 ^c^	
***Women***	**Non-obese**	3520	63.9	43.5 ± 18.5 ^a^	21.2 ± 2.5 ^a^	28.9 ± 4.5 ^a^	15.0 ± 1.4 ^a^	6.2 ± 1.5 ^a^	73.4 ± 8.0 ^a^	<0.001 ^†^
	Underlean	46	0.9	43.6 ± 20.5 ^a,b^	20.4 ± 1.0 ^b^	37.5 ± 2.2 ^b^	12.7 ± 0.5 ^b^	7.7 ± 0.8 ^b^	71.5 ± 4.2 ^b^	
	Proportional	1901	32.1	51.7 ± 18.0 ^b^	26.1 ± 2.7 ^c^	38.8 ± 3.1 ^c^	15.9 ± 1.2 ^c^	10.2 ± 1.8 ^c^	86.3 ± 7.8 ^c^	
	Heavy	165	3.0	44.1 ± 15.6 ^a^	33.9 ± 3.8 ^d^	42.1 ± 4.5 ^d^	19.5 ± 1.0 ^d^	14.4 ± 3.1 ^d^	101.2 ± 8.6 ^d^	
***Part B. Age-stratified obesity subtype prevalence.***										
**Sex**	**Age Group** **(Years)**	**n** **(Obese)**	**Underlean** **n (%)**		**Proportional** **n (%)**			**Heavy** **n (%)**		
***Men***	20–29	192	9 (4.5%)		159 (82.0%)			24 (13.5%)		
	30–39	229	2 (0.7%)		206 (90.0%)			21 (9.3%)		
	40–49	316	10 (3.4%)		277 (87.8%)			29 (8.8%)		
	50–59	312	7 (2.3%)		287 (92.4%)			18 (5.3%)		
	60–69	415	29 (6.2%)		384 (92.9%)			2 (1.0%)		
	70–80	433	71 (17.4%)		362 (82.6%)			0 (0.0%)		
***Women***	20–29	170	6 (4.2%)		146 (85.3%)			18 (10.6%)		
	30–39	197	5 (2.7%)		160 (82.0%)			32 (15.2%)		
	40–49	280	9 (3.5%)		232 (83.2%)			39 (13.3%)		
	50–59	418	7 (1.8%)		380 (91.5%)			31 (6.7%)		
	60–69	539	4 (1.1%)		505 (93.4%)			30 (5.5%)		
	70–80	434	10 (2.6%)		414 (94.6%)			10 (2.8%)		

Values are presented as weighted mean ± SD. Obesity: %BF ≥ 25% (men), ≥35% (women) [28]. Underlean: FFMI < P5; Proportional: FFMI P5–P95; Heavy: FFMI > P95 of the 18–59-year reference group [16]. Different superscript letters (a, b, c, d) within each sex indicate statistically significant differences between groups (*p* < 0.05, Bonferroni-corrected pairwise comparisons following survey-weighted one-way analysis with cluster-robust standard errors). Groups sharing the same superscript letter do not differ significantly. ^†^ Overall *p*-value from survey-weighted 4-group F-test; all variables *p* < 0.001 for both sexes. BMI, body mass index; %BF, percent body fat; FFMI, fat-free mass index; FMI, fat mass index; WC, waist circumference; SD, standard deviation. Obesity: %BF ≥ 25% (men), ≥35% (women) [28]. Underlean: FFMI < P5; Proportional: FFMI P5–P95; Heavy: FFMI > P95 of the 18–59-year reference group. Percentages are survey-weighted prevalence within each age- and sex-specific obese subgroup. FFMI thresholds: Men: P5 = 16.0 kg/m^2^, P95 = 22.2 kg/m^2^; Women: P5 = 13.2 kg/m^2^, P95 = 18.5 kg/m^2^ [16]. BMI, body mass index; %BF, percent body fat; FFMI, fat-free mass index.

## Data Availability

The KNHANES IX datasets analyzed in this study are publicly available from the KDCA website (https://knhanes.kdca.go.kr, accessed on 1 March 2025).

## Supplementary Materials

The following supporting information can be downloaded at https://www.mdpi.com/article/10.3390/nu18081170/s1. Figure S1: Flow Diagram.

## Abbreviations

The following abbreviations are used in this manuscript:

BIA	Bioelectrical impedance analysis
BMI	Body mass index
BFM	Body fat mass
DXA	Dual-energy X-ray absorptiometry
ECW	Extracellular water
FFM	Fat-free mass
FFMI	Fat-free mass index
FMI	Fat mass index
KDCA	Korea Disease Control and Prevention Agency
KNHANES	Korea National Health and Nutrition Examination Survey
KSSO	Korean Society for the Study of Obesity
PhA	Phase angle
SD	Standard Deviation
TBW	Total body water
%BF	Percent body fat

## References

[1] Nuttall F.Q. (2015). Body mass index: Obesity, BMI, and health: A critical review. Nutr. Today.

[2] Korean Society for the Study of Obesity (KSSO) (2023). Clinical Practice Guidelines for Obesity 2023.

[3] Wells J.C.K. (2000). A Hattori chart analysis of body mass index in infants and children. Int. J. Obes. Relat. Metab. Disord..

[4] Prentice A.M., Jebb S.A. (2001). Beyond body mass index. Obes. Rev..

[5] Garn S.M., Leonard W.R., Hawthorne V.M. (1986). Three limitations of the body mass index. Am. J. Clin. Nutr..

[6] Gao Q., Mei F., Shang Y., Hu K., Chen F., Zhao L., Ma B. (2021). Global prevalence of sarcopenic obesity in older adults: A systematic review and meta-analysis. Clin. Nutr..

[7] VanItallie T.B., Yang M.U., Heymsfield S.B., Funk R.C., Boileau R.A. (1990). Height-normalized indices of the body’s fat-free mass and fat mass: Potentially useful indicators of nutritional status. Am. J. Clin. Nutr..

[8] Cruz-Jentoft A.J., Bahat G., Bauer J., Boirie Y., Bruyère O., Cederholm T., Cooper C., Landi F., Rolland Y., Sayer A.A. (2019). Sarcopenia: Revised European consensus on definition and diagnosis. Age Ageing.

[9] Lim Y.S., Yap P.M., Lim S.C., Lee Y.S., Lau K.M., Pang W.W., Chew S.T., Lim J.P., Ismail N.H., Ding Y.Y. (2021). Relationship of fat mass index and fat free mass index with body mass index and association with function, cognition and sarcopenia in pre-frail older adults. Front. Endocrinol..

[10] Baumgartner R.N. (2000). Body composition in healthy aging. Ann. N. Y. Acad. Sci..

[11] Briand M., Raffin J., Gonzalez-Bautista E., Ritz P., Abellan Van Kan G., Pillard F., Faruch-Bilfeld M., Guyonnet S., Dray C., Vellas B. (2025). Body composition and aging: Cross-sectional results from the INSPIRE study in people 20 to 93 years old. GeroScience.

[12] Bosy-Westphal A., Müller M.J. (2015). Identification of skeletal muscle mass depletion across age and BMI groups in health and disease—There is need for a unified definition. Int. J. Obes..

[13] Hattori K. (1991). Body composition and lean body mass index for Japanese college students. J. Anthropol. Soc. Nippon.

[14] Hattori K., Tatsumi N., Tanaka S. (1997). Assessment of body composition by using a new chart method. Am. J. Hum. Biol..

[15] Wells J.C.K., Davies P.S.W., Fewtrell M.S., Cole T.J. (2020). Body composition reference charts for UK infants and children aged 6 weeks to 5 years based on measurement of total body water by isotope dilution. Eur. J. Clin. Nutr..

[16] Guo B., Wu Q., Gong J., Xu H. (2017). Age- and sex-dependent values of the distribution of body composition parameters among Chinese children using the Hattori chart. J. Clin. Desitom..

[17] Wickramasinghe V.P., Lamabadusuriya S.P., Cleghorn G.J., Davies P.S.W. (2012). Hattori chart based evaluation of body composition and its relation to body mass index in a group of Sri Lankan children. Indian J. Pediatr..

[18] Hattori K., Tahara Y., Moji K., Aoyagi K., Furusawa T. (2004). Chart analysis of body composition change among pre- and postadolescent Japanese subjects assessed by underwater weighing method. Int. J. Obes. Relat. Metab. Disord..

[19] Kim C.H., Chung S., Kim H., Park J.H., Kim H.Y., Han K.H., Park Y.S., Kim C. (2011). Norm references of fat-free mass index and fat mass index and subtypes of obesity based on the combined FFMI–%BF indices in the Korean adults aged 18–89 yr. Obes. Res. Clin. Pract..

[20] Pratt J., Narici M., Boreham C., De Vito G. (2025). Dual-energy X-ray absorptiometry derived body composition trajectories across adulthood: Reference values and associations with body roundness index and body mass index. Clin. Nutr..

[21] Batsis J.A., Mackenzie T.A., Lopez-Jimenez F., Bartels S.J. (2018). Sarcopenic obesity in older adults: Aetiology, epidemiology and treatment strategies. Nat. Rev. Endocrinol..

[22] Donini L.M., Busetto L., Bischoff S.C., Cederholm T., Ballesteros-Pomar M.D., Batsis J.A., Bauer J.M., Boirie Y., Cruz-Jentoft A.J., Dicker D. (2022). Definition and diagnostic criteria for sarcopenic obesity: ESPEN and EASO consensus statement. Clin. Nutr..

[23] Luo Y., Wang Y., Tang S., Xu L., Zhao X., Han M., Liu Y., Xu Y., Han B. (2024). Prevalence of sarcopenic obesity in the older non-hospitalized population: A systematic review and meta-analysis. BMC Geriatr..

[24] Coin A., Sergi G., Minicuci N., Giannini S., Barbiero E., Manzato E., Pedrazzoni M., Minisola S., Rossini M., Del Puente A. (2008). Fat-free mass and fat mass reference values by dual-energy X-ray absorptiometry (DEXA) in a 20–80 year-old Italian population. Clin. Nutr..

[25] Ling C.H., de Craen A.J., Slagboom P.E., Gunn D.A., Stokkel M.P., Westendorp R.G., Maier A.B. (2011). Accuracy of direct segmental multi-frequency bioimpedance analysis in the assessment of total body and segmental body composition in middle-aged adult population. Clin. Nutr..

[26] Schutz Y., Kyle U.U.G., Pichard C. (2002). Fat-free mass index and fat mass index percentiles in Caucasians aged 18–98 y. Int. J. Obes..

[27] Ofenheimer A., Breyer-Kohansal R., Hartl S., Guenther G., Gindlhumer O., Galler B.C., Geroushi A., Stelzmacher L., Brath H., Baumgartner J. (2020). Reference values of body composition parameters and visceral adipose tissue (VAT) by DXA in adults aged 18–81 years—Results from the LEAD cohort. Eur. J. Clin. Nutr..

[28] Seino S., Shinkai S., Iijima K., Obuchi S., Fujiwara Y., Yoshida H., Kawai H., Nishi M., Murayama H., Taniguchi Y. (2015). Reference values and age differences in body composition of community-dwelling older Japanese men and women: A pooled analysis of four cohort studies. PLoS ONE.

[29] Soh B.P., Lee S.Y., Wong W.Y., Koh A.S., Hartman M., Ding Y.Y., Lim W.S. (2022). Body composition reference values in Singaporean adults using dual-energy X-ray absorptiometry—The Yishun study. PLoS ONE.

[30] Jin M., Du H., Zhang Y., Zhu H., Xu K., Yuan C., Wang W., Li L. (2019). Characteristics and reference values of fat mass index and fat free mass index by bioelectrical impedance analysis in an adult population. Clin. Nutr..

[31] Wells J.C.K. (2014). Toward body composition reference data for infants, children, and adolescents. Adv. Nutr..

[32] Hong S., Oh H.J., Choi H., Kim J.G., Lim S.K., Kim E.K., Pyo E.Y., Oh K., Kim Y.T., Wilson K. (2011). Characteristics of body fat, body fat percentage and other body composition for Koreans from KNHANES IV. J. Korean Med. Sci..

[33] Kim K., Yun S.H., Jang M.J., Oh K.W. (2013). Body fat percentile curves for Korean children and adolescents: A data from the Korea National Health and Nutrition Examination Survey 2009–2010. J. Korean Med. Sci..

[34] Park J.M., Lee J., Kim Y., Won C.W., Kim Y.J. (2015). Reference values of body composition indices: The Korean National Health and Nutrition Examination Surveys. Yonsei Med. J..

[35] Korea Disease Control and Prevention Agency (KDCA) (2024). The 9th Korea National Health and Nutrition Examination Survey (KNHANES IX) User Guide.

[36] Gallagher D., Heymsfield S.B., Heo M., Jebb S.A., Murgatroyd P.R., Sakamoto Y. (2000). Healthy percentage body fat ranges: An approach for developing guidelines based on body mass index. Am. J. Clin. Nutr..

[37] Sowers M., Zheng H., Tomey K., Karvonen-Gutierrez C., Jannausch M., Li X., Yosef M., Symons J. (2007). Changes in body composition in women over six years at midlife: Ovarian and chronological aging. J. Clin. Endocrinol. Metab..

[38] Jang H., Kim R., Lee J., Lee D., Giovannucci E.L., Oh H. (2023). Overall and abdominal obesity and risks of all-cause and cause-specific mortality in Korean adults: A pooled analysis of three population-based prospective cohorts. Int. J. Epidemiol..

[39] Chen L.K., Woo J., Assantachai P., Auyeung T.W., Chou M.Y., Iijima K., Jang H.C., Kang L., Kim M., Kim S. (2020). Asian Working Group for Sarcopenia: 2019 Consensus update on sarcopenia diagnosis and treatment. J. Am. Med. Dir. Assoc..

