# Supplementary figures and images for: National Reference Values of FFMI and FMI Using Body Composition Chart in Korean Adults
Source: Nutrients. 2026 Apr 8;18(8):1170. doi: 10.3390/nu18081170 (PMC13118327; doi:10.3390/nu18081170)

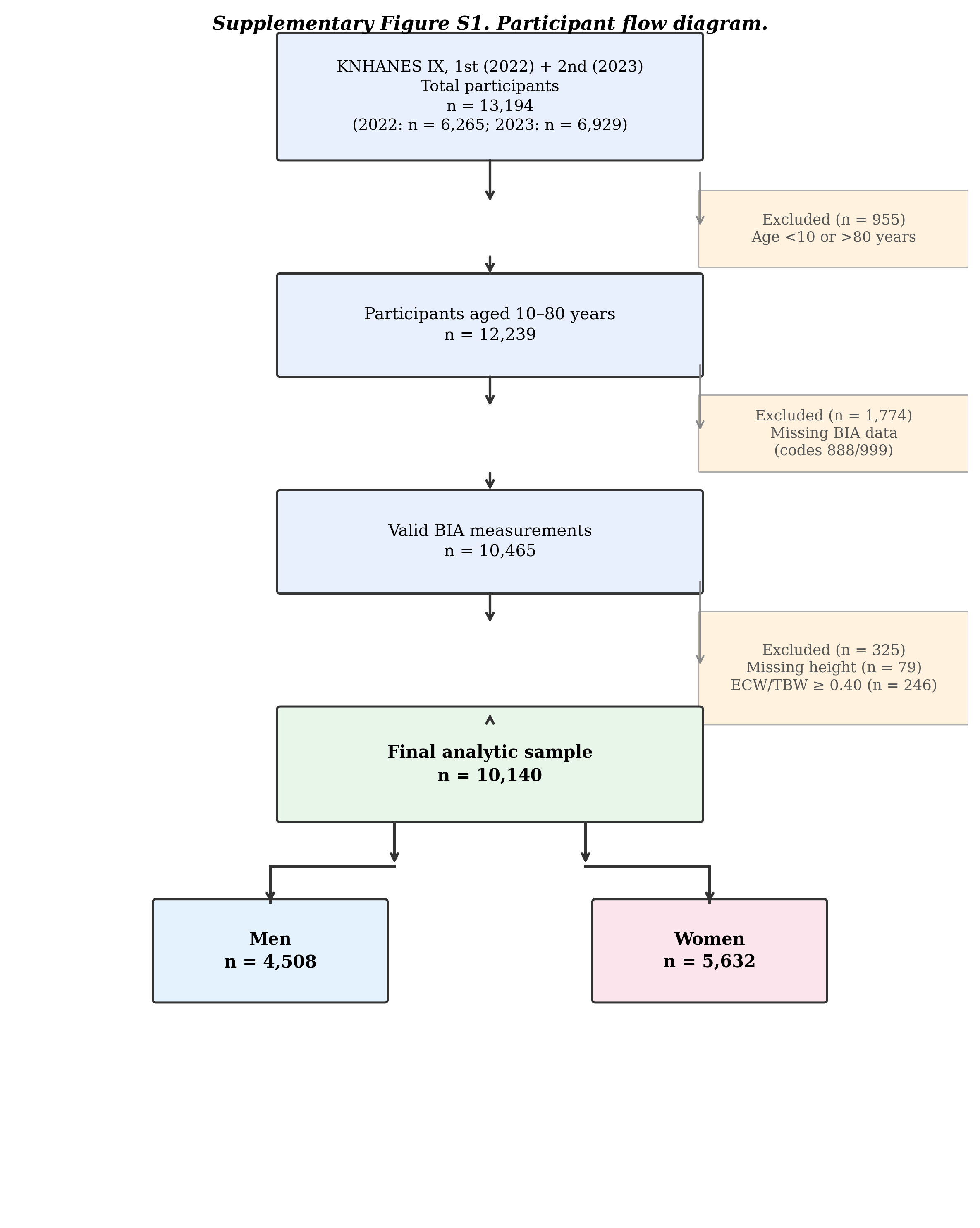

Supplement: Supplementary file 1 [file nutrients-18-01170-s001.zip › nutrients-4236941-supplementary.png]
